# The Keap1 signaling in the regulation of HSP90 pathway

**DOI:** 10.1007/s12192-022-01253-5

**Published:** 2022-04-01

**Authors:** Angela Bonura, Miriam Giacomarra, Giovanna Montana

**Affiliations:** 1 Consiglio Nazionale delle Ricerche (CNR), Istituto di Ricerca e Innovazione Biomedica, Via Ugo La Malfa 153, 90146 Palermo, Italy; 2 Consiglio Nazionale delle Ricerche (CNR), Istituto di Farmacologia Traslazionale, 00133 Roma, Italy

**Keywords:** HSP90, Keap1, COX2, Oxidative stress, Ferulic acid

## Abstract

The Keap1 protein is the master modulator of Nrf2 pathway; moreover, it is the hub of such important processes as cancer, cell stress, inflammation, and chemio- and radio-resistance. That is why Keap1 has become an intriguing pharmacological target. Many recent data show that Keap1 interacts with HSP90 protein. In this study, we use ferulic acid (FA) as antioxidant and anti-inflammatory agent, able to relieve inflammatory response. It is known that treatment with 100 μg of FA can significantly decrease the oxidative stress, so it turns to be useful to study the antioxidant regulation. The RAW 264.7 cells transfected with si-Keap1 and LPS treated are the in vitro model used to study the effects of Keap1 silencing on HSP90 activities and the FA antioxidant modulation. Immunoblot data and qPCR analysis show that Keap1 is involved in HSP90 modulation and on anti-oxidative response. Keap1 silencing affects negatively COX2 activation; in fact western blot and qPCR analysis conducted on RAW 264.7 cells Keap1silenced highlight that LPS treatment does not induce COX2 activation. In addition, the FA anti-oxidative and modulatory effect is abolished in COX2 pathway. The same results are point out using human A549 cell line with an allelic mutation on Keap1 gene, and the protein results are partially inactive. This preliminary study points out that Keap1protein is involved in HSP90 and anti-oxidative pathway regulation.

## Introduction

Heat shock proteins (HSPs) are highly conserved and constitutively expressed molecules in the cell. They have been studied for a long time and were initially related mostly to heat stress (Geraci et al. 2003, 2004), although it soon became clear that they played many other roles. HSPs usually constitute about 5–10 % of the total protein in most cells, but their intracellular concentrations can be increased by stressors, e.g., increased temperature (fever), oxidative stress, ethanol, and infection that induce protein unfolding, misfolding, or aggregation (Bukau et al. 2016; Kriegenburg et al. 2012). They act as molecular chaperones or proteases and are localized in the cytoplasm and in various intracellular compartments. Recently, many studies have shown that there is a connection between Hsp90, inflammation, and cancer. Therefore, this chaperone has gained a particular position at the core of the scientific interest (Tukaj and Węgrzyn 2016). Hsp90 is a highly abundant molecular chaperone, essential for cell growth and survival; it regulates the function of various proteins including several protein kinases and transcription factors (Neckers and Ivy 2003; Chen et al. 2002). Inhibition of Hsp90 by using small-molecule inhibitors has been extensively studied for its therapeutic potential in targeting cancer cells and promoting apoptosis (Bucci et al. 2000). Hsp90 participates in stabilizing and activating more than 200 “client” proteins, including key signaling molecules such as nuclear transcription factors NF-κB, STATs, p53, and kinases (e.g., Raf/MEK/ERK, PI3K/AKT, and p38/ MAPK) (Edwards and Basler 2015). Thus, it regulates crucial cellular processes, e.g., inflammation, growth, survival, differentiation, and apoptosis (Trepel et al. 2010). Inflammation is a very complex and interconnected phenomenon, so the observation that the proteotoxic pathway guided by HSP90 interacts with the antioxidant one is very interesting. Inflammation causes the onset of oxidative stress and the activation of cellular signaling pathways like the one concerning the nuclear factor erythroid-derived 2-like 2 (NRF2) (Lin et al. 2019). Nrf2 is a member of a family of basic leucine transcription factors that binds to the promoter region of genes involved in redox regulation, proteostasis, DNA repair, prevention of apoptosis, iron and heme metabolism, and phase I, II, and III drug/xenobiotic metabolism (Keum and Choi 2014). The activation of this transcriptional factor is controlled at transcriptional and post-transcriptional level (Nguyen et al. 2004; McMahon et al. 2010). In response to different stimuli, Nrf2 is stabilized and moves to the nucleus where it activates the transcription of its target genes. Nrf2 is a modular protein that presents seven domains of homology to Nrf2-ECH (Neh), each of which performs different functions; in particular, the Neh2 domain interacts with Keap1 (Itoh et al. 1995; McMahon et al. 2004). Under physiological conditions, Keap1protein maintains low Nrf2 levels intracellular levels, having a half-life of 10–30 min. In conditions of oxidative stress, Keap1 is oxidized on the reactive and inactivated cysteine residues, while Nrf2 stabilizes and moves into the nucleus (Zhang and Hannink 2003). The Nrf2-sMaf complex binds, in a specific sequence manner, to the antioxidant response elements (ARE 5′-TGACXXXGC-3′) in the promoter region of the target genes (Dinkova-Kostova et al. 2015). The role of Keap1 in the regulation of Nrf2 lifetime is now well known, and it is also clear that many anti-inflammatory agents, especially ferulic acid, flavonoids, hydroxycinnamic acids, and tannins, are able to reduce the oxidative stress levels (Srinivasan et al. 2007). Our previous study carried out in RAW264.7 cells treated with LPS showed that the anti-inflammatory activity of ferulic acid depends on Keap1 (Giacomarra et al. 2020). HSP 90 and Nrf2 are the master regulators of cellular homeostasis under stressful conditions, and many studies put in evidence that Hsp90 and KEAP1 interact upon heat shock, leading to the activation of NRF2 (Niture et al. 2010), and that the environmental redox changes can induce heat shock genes (Liu et al. 2016). KEAP1/NRF2 pathway and the heat shock response are cytoprotective pathways that are trigged by a stress signal in the cellular environment (Talalay et al. 1988; McMahon et al. 2010; Zhang et al. 2011). So in this paper, we discuss results preliminary, but very intriguing: we analyze the effect of Keap1 silencing on HSP90 pathway in an in vitro model RAW264.7 cells treated with LPS and in A549, a Keap1 mutated adenocarcinoma cell line (Singh et al. 2006).

## Materials and methods

### Cell culture and reagents

The mouse macrophage-like virus-transformed leukemia cell line RAW 264.7 was purchased from American Type Culture Collection (ATCC). RAW 264.7 cells and A549 cell line (provided by Francesca Sardina e Cinzia Rinaldo (IBPM-CNR, Roma, Italy) were cultured in Dulbecco’s Modified Eagle Medium (DMEM) supplemented with 10% v/v heat-inactivated fetal bovine serum (FBS) and antibiotics (100 U/ml penicillin and 100 μg/ml streptomycin) at 37°C in a humidified atmosphere with 5% CO_2_. FBS, DMEM, penicillin, and streptomycin (10,000 U/ml) were purchased from GIBCO (Grand Island, NY). LPS from *E. coli* serotype O55:B5 and ferulic acid (FA; CAS Number: 537-98-4) were purchased from Sigma-Aldrich, Inc. (St. Louis, MO). TRIzol was purchased from Invitrogen, the QuantiNova RT PCR kit from Hilden, Germany, and BrightGreen 2X qPCR MasterMix-ROX from abm (Canada). Nitrocellulose blotting membrane was purchased from Amersham Protran (Buckinghamshire, UK).

### RT-qPCR

RAW 264.7 cells were cultured (1 ×10^6^ cells/well) in a 6-well plate overnight. Cells were treated with 100 ng/ml LPS or without (negative control) in the presence or absence of 100 μM ferulic acid in DMEM supplemented with 10% bovine serum for 4 h. Cells stimulated with 100 ng/ml LPS for 4 h served as a positive control. After 4 h of stimulation, the cells were detached from the wells and washed once with PBS. Total RNA was isolated with TRIzol according to the manufacturer’s instructions and was quantified by UV absorbance spectrophotometry and reverse transcribed with QuantiNova RT PCR kit. QPCR was performed in triplicate on each cDNA sample for each gene using BrightGreen 2X qPCR MasterMix-ROX:

- Hsp90 NM005348.2 F: 5′-CGATGAATATGCCATGACT-3′
  - R: 5′-TCCATAGCAGATTCTCCAG-3′
- COX2 GI:31981524 F:5′-CAGACAACATAAACTGCGCCTT-3′
  - R: 5′-GATACACCTCTCCACCAATGACC -3′
- HPRT NM194 F:5′-GCTATAAATTCTTTGCTGACCTGCTG-3′
  - R: 5′-AATTACTTTTATGTCCCCTGTTGACTGG-3′

by using primers set Quantitect from Qiagen. The threshold cycle (CT) values were calculated against the housekeeping gene Hprt. In order to report of results, all data were normalized to Hprt, which was assigned an arbitrary expression level of 10,000, and relative gene expression values were calculated by the following formula: relative expression 10,000/2 CT, where CT (gene CT/Hprt CT). Melt curve analysis was conducted to verify the purity and size of the resultant PCR products. At least three distinct biological samples were examined for each gene and treatment (each one performed in triplicate).

### siRNA transfection

The RAW264.7 cells (5 × 105 cells/well) were seeded in 6-well plates for 24 h. Briefly, the siRNA pool for Keap1 (Qiagen) and NC-siRNA (Qiagen) were incubated with Lipofectamine RNAiMAX (Promega) in basal media with no serum or antibiotics and allowed to complex for 10 min at room temperature. Then, the complex was added to the cell suspension of each well (final siRNA pool concentration of 10 nM). Finally, cells were incubated for 24 h in a humidified incubator and then used for the analysis.

### Western blotting

RAW 264.7 cells (1×107 cells) were cultured in 10-cm dishes (Falcon) and allowed to adhere for 24 h. After treatment with FA 100μM 1 followed by co-incubation with LPS 100 ngr/ml for 4 h, the cells were washed twice with cold PBS. Whole-cell lysates were obtained using RIPA buffer (Cell Signaling Inc. Beverly, MA, USA). The protein concentration of cell lysates was determined by the Bradford method. An amount of protein (30 μg) was separated on 8–16% Tris-Glycine Gel (BioRad) gels by electrophoresis and transferred to a nitrocellulose membrane. The membranes were subsequently incubated for 1 h at room temperature with 3% BSA in TBS buffer (0.1% v/v) to block non-specific binding and incubated with an appropriate primary antibody in 1% BSA in TBST (tween 0.01% v/v). Antibodies polyclonal anti-mouse recognizing HSP90, lamin B1, Keap1, COX2, and β-actin were purchased from Santa Cruz Biotechnology (Santa Cruz, CA, USA). Incubation with the secondary antibodies Alexa Fluor 680 goat anti-rabbit (1:2000) and Alexa Fluor 800 rabbit anti-mouse (1:5000) (Molecular Probes, Life Technologies, Carlsbad, CA, USA) was performed for 1 h at room temperature. Densitometry analysis was conducted using the Odyssey Infrared Imaging System (Li-COR Bioscience, NE, USA).

### Statistics

#### Statistical analysis

All data were analyzed by the one-way analysis of variance (one-way ANOVA) compared with the respective control group, followed by the multiple comparison test of Tukey’s, using the OriginPro 7.5 statistical program with the level of significance set to *P* < 0.05. Each result is reported as the mean of three independent replicate experiments ±SE.

## Results

### siRNA-mediated Keap1 knockdown alters HSP90 activity in RAW264.7 cells

**Figure 1:**
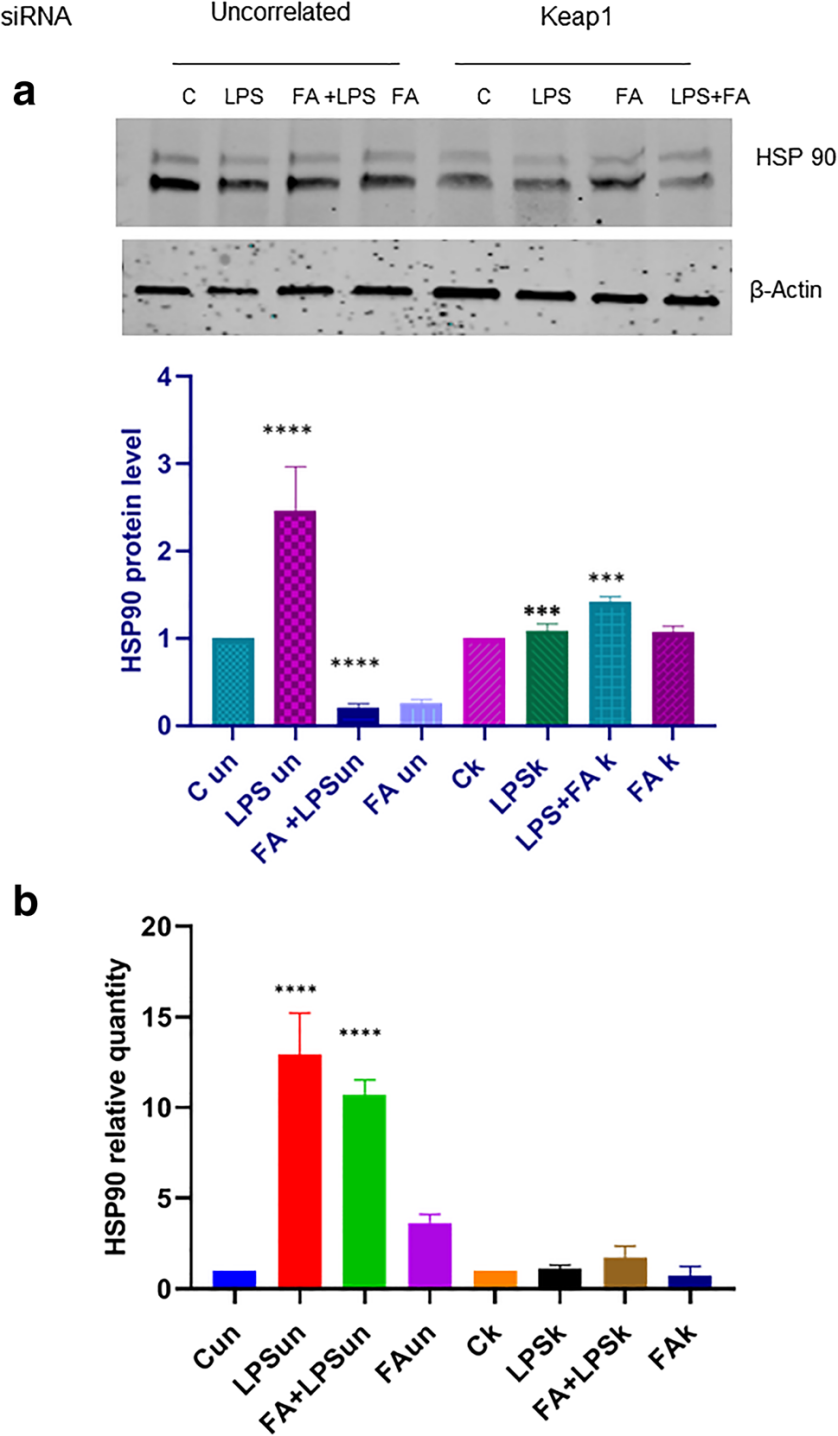
a Immunoblot analysis of HSP 90 in RAW264.7 cells transfected with uncorrelated (un) and with si-Keap1 RNA (k). RAW264.7 cells transfected with si-Keap1were treated with or without FA 100 μM for 1 h and then treated with LPS 100 ngr/ml for 4 h like the uncorrelated siRNA-transfected RAW264.7. The whole-cell lysates were obtained and analyzed with anti-HSP90 antibody (C-20, Santa Cruz Biotechnology). Representative immunoblotting shows results for the protein levels of HSP 90. Histogram is representative of the means ± SD of three replicates after normalization with actin. Protein levels are reported in arbitrary units as fold increase or decrease compared to controls that are set to 1. Asterisks (*) indicate significant differences among groups (* *p* < 0.05, ** *p* < 0.01, *** *p* < 0.001), ****p< 0.0001. **b** HSP90 mRNA expression in uncorrelated RNA (un) and siKeap1-transfected RAW 264.7 cells (k). RAW 264.7 cells were treated as described above. RNA was extracted, and cDNA was analyzed by qPCR with hsp90 primers. Non-stimulated cells worked as a negative control. The graph shows the level of hsp90 mRNA expression. Results do not show significant changes in hsp 90 expression in siKeap1-transfected RAW 264.7 cells (k). Levels are expressed in arbitrary units as fold increase compared to controls assumed as 1. The data shown represents three independent experiments, each of which performed in triplicate; asterisks (*) indicate significant differences among groups (****p< 0.0001).

Heat shock proteins (HSPs) are molecular chaperones produced in response to oxidative stress (OS), and there is evidence implicating heat shock protein 90 as a mediator of activation by bacterial lipopolysaccharide. In order to assess if Keap1-Cullin 3 pathway is related with HSP90 activity, we transfected the macrophage-like RAW 264.7 cells with siRNAs for Keap1 and uncorrelated siRNA. Moreover, immunoblot analysis was conducted to verify the protein level of HSP90; 24 h after transfection, cells were exposed to 100 ng/ml LPS for 4 h, and 1 h before LPS, the cells were treated or not with 100 μM FA. Several studies have already pointed out that 100 μM is a non-cytotoxic and antioxidant useful dose of FA to modulate the oxidative stress induced by LPS. Total protein extracts were prepared and analyzed in immunoblotting for Hsp90. Figure 1a shows that in RAW 264.7 cells transfected with uncorrelated siRNA, the Hsp90 level protein increases after LPS treatment, and FA is able to reduce such increase; instead, in the siRNA-mediated Keap1 knockdown, the FA modulation on LPS inducted HSP90 increase activity does not occur. Another important aspect was deeply focused on the regulation of hsp90a transcription. In the same system, we have also analyzed the expression of hsp90 mRNA to investigate the effects of siRNA-mediated Keap1 knockdown on activation of the hsp90 mRNA transcription: the qPCR analysis highlights that in RAW 264.7 cells transfected with uncorrelated siRNA, LPS significantly triggers the hsp90 mRNA transcription, and FA treatment reduces the mRNA level (Fig. 1b). On the contrary, in RAW 264.7 cells transfected with siRNAs for Keap1, LPS is not able to induce an increase of hsp90 mRNA level, despite the LPS activation. These results disclose an unrecognized mechanism which contributes to connect Keap1 and HSP90.

### HSP90 regulation of anti-oxidative pathway is Keap1-mediated

**Figure 2:**
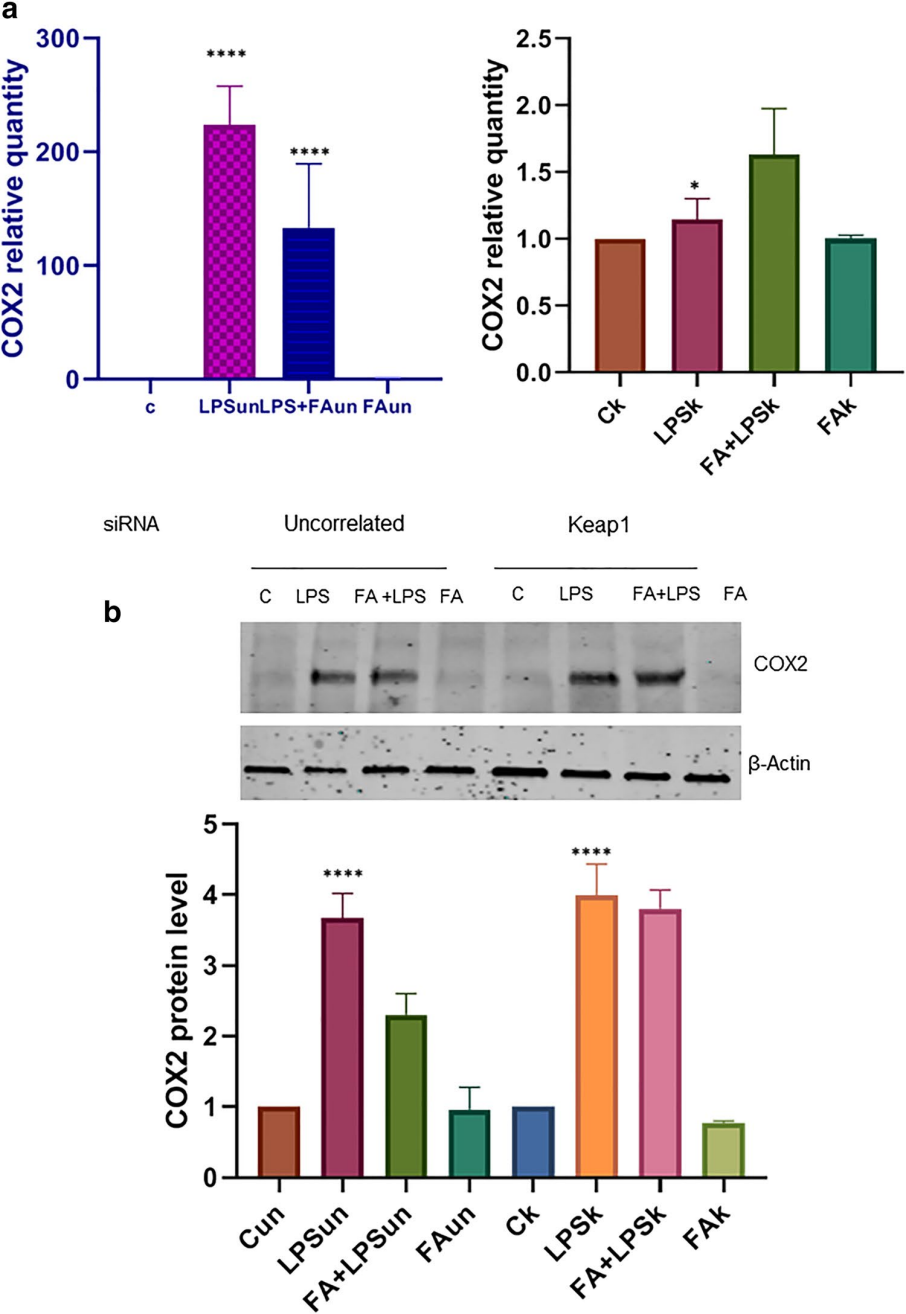
**a** QPCR analysis of COX2 mRNA expression in RAW 264.7 cells transfected with uncorrelated (un) and siKeap1RNA (k). RAW 264.7 cells were transfected and after 24 h cells were pre-treated with or without FA 100 μM for 1h and then treated with LPS 100 ngr/ml for 4h; total RNA was extracted, and cDNA was analyzed by qPCR. Non-stimulated cells worked as control. Significant change in mRNA expression are showed in the graph at the left (RAW 264.7 cells transfected with uncorrelated RNA). Levels in the graph are expressed in arbitrary units as fold increase compared to controls assumed as1, using the endogenous gene HPRT for normalization. The data shown represents three independent experiments, each of which performed in triplicate. Asterisks (*) indicate significant differences among groups (* *p* < 0.05, **** *p* < 0.0001). **b** Immunoblot analysis of COX2 protein in RAW264.7 cells transfected with uncorrelated and si-Keap1 RNA. RAW 264.7 cells were treated as described above. Total proteins were extracted, and immunoblot analysis of COX2 protein level was effectuated. Here, a representative image of the protein levels obtained from three replicates after normalization with actin is reported. Protein levels are reported in arbitrary units as fold increase or decrease compared to controls that are set to 1. Asterisks (*) indicate significant differences among groups (**** *p* < 0.0001).

HSP90—as a molecular chaperone—supports the active conformational structure and function of several signal proteins, termed “client” proteins; some of them are involved in cancer and inflammation. The COX-2 is known as prostaglandin-endoperoxide synthase (PTGS), an enzyme which is responsible for the formation of key biological mediators such as prostanoids (prostaglandins, prostacyclin, and thromboxane). It exists as two distinct isoforms (Kraemer, Hinz). COX-2 is inducible and expressed by cells that are involved in inflammatory processes. We used the RAW264.7 cells transfected with siRNA Keap1 to study the COX2 activity and evaluate the HSP90 regulation on this activity. QPCR analysis of COX2 mRNA expression showed in Figure 2a indicates that the level of COX2 mRNA does not increase in RAW264.7 cells Keap1 silencing unlike the uncorrelated RNA-transfected cells, despite LPS stimulus occurs. The results emerged from immunoblot analysis (Fig. 2b) put in evidence that FA does not modulate the COX2 protein activity when Keap1 is inactivated. Therefore, these results imply that the HSP90 regulation on anti-oxidative pathway is Keap1-mediated.

### Study of HSP90 activity in A549 cell line

**Figure 3:**
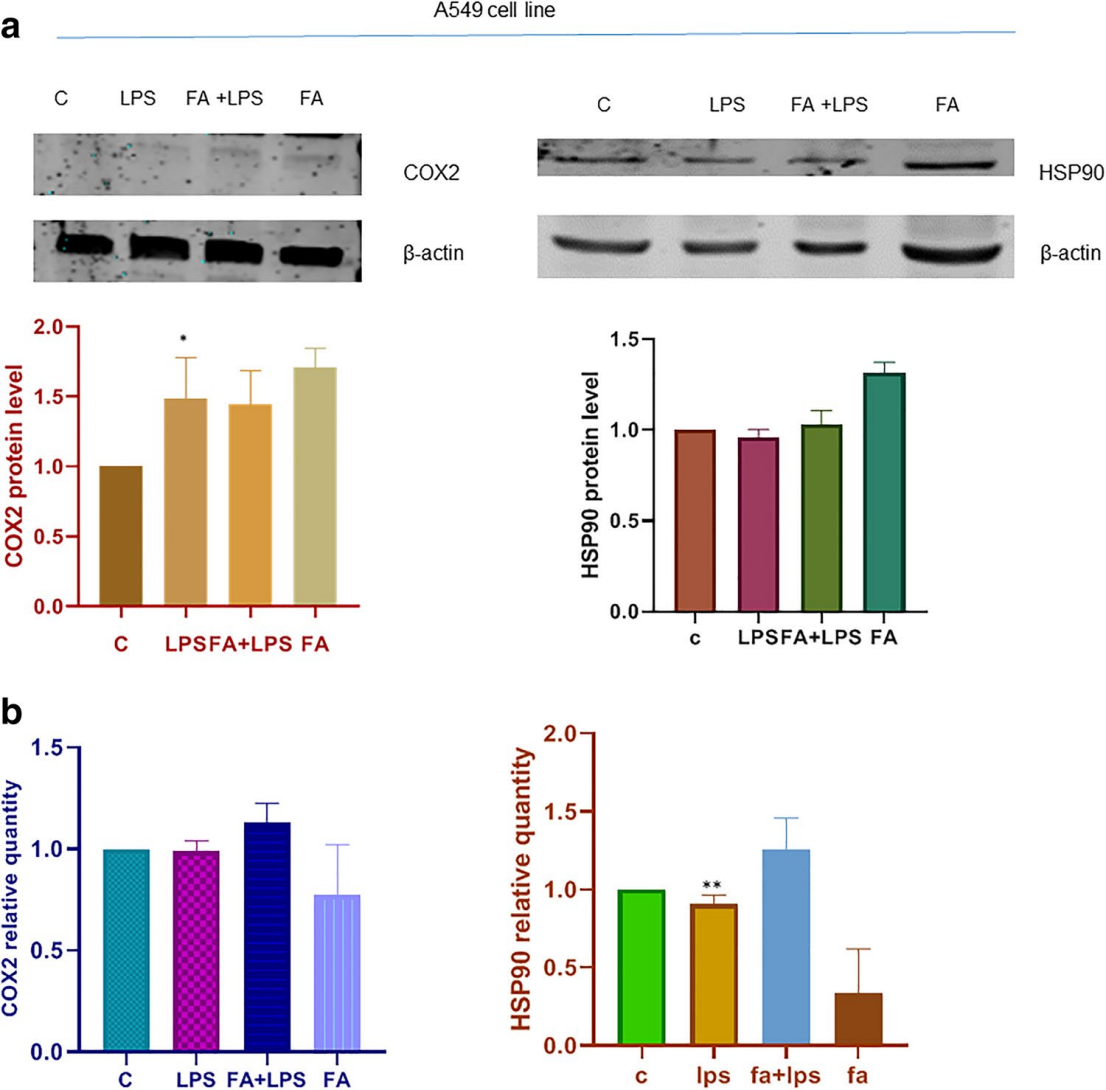
**a** Immunoblot analysis of HSP90 and COX2 proteins in A549 cell line. A549 cells were pre-treated with or without FA 100μM for 1h and then with LPS 100ngr/ml for 4h. Total proteins were extracted, and immunoblot analysis of HSP 90 and COX2 protein level was effectuated. Representative immunoblotting results show limited changes on the protein level. Histogram is representative of the means ± SD of three replicates after normalization with actin. Protein levels are reported in arbitrary units as fold increase or decrease compared to controls that are set to 1. Asterisks (*) indicate significant differences among groups (* *p* < 0.05). **b** HSP90 and COX2 activities are altered in A549 cell line. Expression levels of HSP90 and COX2 mRNA in A 549 cells. The cells were treated as above. Total RNA was extracted, and cDNA was analyzed by qPCR. Levels are expressed in arbitrary units as fold increase compared to controls assumed as1, using the endogenous gene HPRT. The data shown represents three independent experiments. Asterisks (*) indicate significant differences among groups (** *p* < 0.01).

A549 cells are adenocarcinomic human alveolar basal epithelial cells carrying a homozygous Keap1 mutation (G333C) that alters binding with Nrf2. We used the A549 lung cancer cell line to verify that HSP90 regulation on COX2 requires active Keap1. Total protein and RNA were extracted from A549 cells exposed to 100 ng/ml LPS for 4 h and 1 h before LPS the cells were treated or not with 100 μM FA. Immunoblot and qPCR analyses were conducted. The analysis of HSP90 and COX2 level protein is shown in Figure 3a: the differences of HSP90 protein level in each group are not significant, and the same can be highlighted in immunoblot for COX2 protein. These data are confirmed by qPCR analysis of mRNA both for Hsp 90 and COX2. The amplification of cDNA with specific primers for Hsp90, COX2, and HPRT as internal standard showed that the transcription of HSP90 and COX2 mRNA does not increase also in LPS treated cells. These data indicate that in A549 cells in which Keap1 has an inactivating mutation, HSP90 and antioxidant pathways are altered. It can be assumed that Keap1 is an important redox-sentinel, and its function is necessary to activate properly the anti-oxidative pathway.

## Discussion

**Figure 4:**
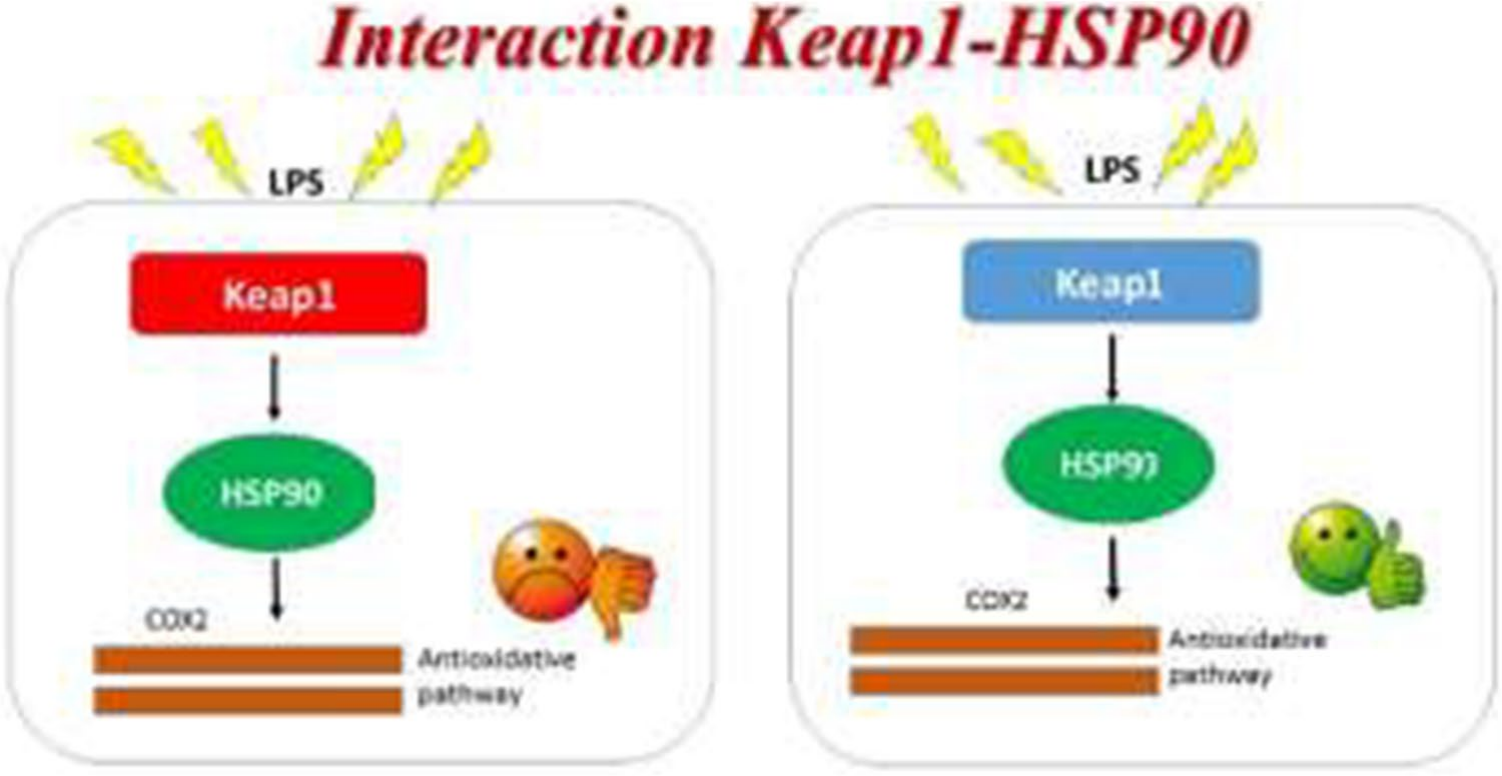
Schematic representation of the interaction between Keap1 protein and HSP90 protein. In homeostatic condition, Keap1 is able to interact with HSP90 protein and promotes the antioxidant response when an oxidant stimulus occurs. Alterations in Keap1 functionality, as an example in A549 cell line or when Keap1 is knockdown with siKeap1 RNA transfection, negatively affect the COX2 activity.

The two most important molecular pathways involved in the antioxidant defense and in the cytoprotective activities are the Nrf2/Keap1 and Heat Shock pathways. In our previous paper (Giacomarra et al. 2020), we highlighted that Keap1 has an important role not only as a modulator of Nrf2, but it is able to modulate the transcription and protein expression of IKKβ, even if the mechanism is not fully clear. In addition, Keap1 is also required for the molecular mechanism underlying the anti-inflammatory effect of ferulic acid. In this study, we demonstrate that Keap1protein plays an important role in the regulation of HSP90 activity. The molecular approach based on silencing of Keap1 in RAW264.7 cells shows that HSP90 activation does not occur; in fact the mRNA expression is deeply altered. We used macrophage-like LPS-stimulated RAW264.7 cells, since such macrophages have vital roles in regulating inflammatory and antioxidant responses and A549—a lung adenocarcinoma cell line—owing to its point mutation in the Keap1 allele (Singh et al. 2006). For this reason, A549 cell line represents a useful model to study the effects of Keap1 loss of functionality. Our data show that when Keap1 is silenced or mutated, the cell is most susceptible to the oxidative damage, in that the anti-oxidative defense is less active, as demonstrated by the lower activation of COX2. COX2 is a very important antioxidant enzyme, and its activation occurs when there are pro-inflammatory conditions (Hinz and Brune 2002; Mohammadi et al. 2016; Kraemer et al. 1992). Our observations suggest a strong cooperative effect between the Keap1 and HSP90 proteins. The cooperativity effect exists because the two proteins are required to activate appropriately COX2 and the reciprocal interactions of HSP90 and Keap1 activities represent a regulatory loop able to influence the response to stressful agents (Fig. 4). The qPCR analysis carried out in Keap1 silenced and LPS treated RAW 264.7 cells showed that the COX2 activation does not occur. The most recent researches have highlighted that Keap1 is not only the repressor of Nrf2 but the most important molecular redox-sensor in the cell (Itoh et al. 2004; Satoh et al. 2006). The scaffold formed by the cysteine-stretch are the nucleus of redox sensor activities. Modifications into the cysteine-stretch in fact alter the Keap1 functionality (Saito et al. 2015). Our data are in line with the literature since they prove that Keap1 protein is required for the cell to receive the stimuli of oxidative stress and then to activate HSP90 protein. The same effect was observed in A549 cells treated with LPS. Many studies point out that Keap1 protein is not only the modulator of Nrf2 pathway but is the crucial point of connection between many pathways, such as the heat shock and antioxidant pathways (Kopacz et al. 2020). The present study is a little contribution to that. Furthermore, the observation that FA is not effective as antioxidant agent in Keap1 silenced RAW264.7 cells—when LPS treatment occurs—is an important scientific evidence, as some previous studies show too (Lampiasi and Montana 2018). It is already proved that Keap1 interacts with IKK chinase (Bloom et al. 2019), and the antioxidant/anti-inflammatory mechanism played by a lot of phenolic natural molecules is mediated by IKK chinase activity (Zeng et al. 2015; Lee et al. 2015). Consequently, we suppose that the fall in the FA effectiveness is linked to the altered interaction between IKK chinase activity and Keap1. As the activity of hsp90a promoter is regulated by NF-κB transcription factors (Ammirante et al. 2008), it can be assumed that the modifications in Keap1protein alter the cell defense machinery. Our results highlight that Keap1 protein is the key point of the linkage between oxidative stress and heat shock response, and we can speculate that in oxidant conditions, Keap1 is the molecular transductor of chemical stimuli into activation of molecular pathway such as the HSP90 protein. We proved that HSP90 activity was strongly inhibited by Keap1 alteration also pro-inflammatory stimuli occurring. These outcomes elucidate the role of Hsp90 as a mechanistic link between inflammation and oxidative stress, since Hsp90 is one of the most important molecular chaperones controlling the client proteins activities and pathways (Hoter et al. 2018; Shukla and  Pitha 2012).
